# A new niclosamide derivatives‐B17 can inhibit urological cancers growth through apoptosis‐related pathway

**DOI:** 10.1002/cam4.1635

**Published:** 2018-06-28

**Authors:** Chia‐Lun Wu, Chun‐Liang Chen, Hsu‐Shan Huang, Dah‐Shyong Yu

**Affiliations:** ^1^ Graduate Institute of Life Science National Defense Medical Center Taipei Taiwan; ^2^ Division of Urology Department of Surgery Tri‐Service General Hospital National Defense Medical Center Taipei Taiwan; ^3^ Graduate Institutes for Cancer Biology and Drug Discovery College of Medical Science and Technology Taipei Medical University Taipei Taiwan

**Keywords:** apoptosis, migratory ability, niclosamide, niclosamide derivatives, urological cancers

## Abstract

The incidence and mortality rate of urological cancers is increasing yearly. Niclosamide has been repurposed as an anti‐cancer drug in recent years. Synthesized derivative of niclosamide was testified for its anti‐cancer activity in urological cancers. MTT assay was used to measure the cytotoxicity effect of niclosamide and its derivatives in urological cancer cell lines. Migratory ability was monitored by scratch migration assay. Apoptosis and cell cycle changes were analyzed by annexin V and PI staining. The apoptosis‐related signal proteins were evaluated by western blotting. T24 had the best drug sensitivity with the lowest IC_50_ in niclosamide and B17 treatment than DU145 and Caki‐1 cells. After niclosamide and B17 treatment, the mitotic cells were decreased, but apoptotic bodies and morphology changes were not prominent in T24, Caki‐1, and DU145 cells. The migratory ability was inhibited in niclosamide treatment than control group on Caki‐1 cells and niclosamide and B17 treatment than control group on DU145 cells. Early apoptosis cells were increased after niclosamide and B17 treatment than control group without cell cycle changes in T24, Caki‐1, and DU145 cells. Programmed cell death was activated majorly through PAPR and bcl‐2 in T24 and caspase‐3 in Caki‐1 cells, respectively. Niclosamide and B17 derivative had good ability in inhibition proliferation and migratory ability in T24, Caki‐1, and DU145 cells without prominent morphology and apoptotic body changes. UCC cells are more sensitive to niclosamide and B17 treatment. Early apoptosis was induced after niclosamide and B17 treatment through different mechanisms in T24, Caki‐1, and DU145 cells.

## 1 INTRODUCTION

Cancer is the pioneer of ten death causes in Taiwan, 92,682 people were first diagnosed with invasive cancer accompanied 42,559 people death in 2011.^1^ 7932 people was belonged to urological cancers, about 8.6% of total invasive cancer accompanied 2408 people deaths (30.4% mortality rate).^1^ Totally 1960, 4628, and 1344 people were first diagnosed with bladder, prostate, and renal cancer and accompanied 790 (40.3% mortality rate), 1096 (23.7% mortality rate), and 522 (38.8% mortality rate) deaths in 2011, respectively.

Approximately 30% of patients with papillary tumors of bladder will progress to invasive urothelial cell carcinoma (UCC), whereas radical cystectomy is the standard therapy.^2^ Unfortunately, this disease recurs in up to 50% of these patients despite surgery, and is potentially lethal. Half of the patients with muscle‐invasive urinary bladder carcinoma will develop into metastatic disease.^3^ GC (gemcitabine and cisplatin) and MVAC (methotrexate, vinblastine, adriamycin (doxorubicin), and cisplatin) have been the standard systemic chemotherapy in advanced urothelial carcinoma. These regimens have shown significant response rates in this patient population. Nevertheless, disease does recur with most patients who unfortunately do succumb to the disease.^4^ Meanwhile, for patients with renal cell carcinoma (RCC), approximately 30% patients will progress to metastasis after first diagnosed.^5^ Targeting angiogenetic factors from the VEGF family has become an effective strategy to inhibit tumor growth. Despite the initial enthusiasm, the angiogenesis inhibitors showed only moderate survival benefit as monotherapy, along with a high cost and many side effects.^6^ Regarding castration‐resistant prostate cancer (CRPC), the standard first‐line treatment is docetaxel‐based chemotherapy. However, CRPC may not respond to docetaxel due to drug resistance or other causes.^7^ Hence, develop new anti‐cancer drug is still urgent needed for these common urological cancers.

However, drug development is very expensive and long period from the initial lead discovery to the final medication.^8^ Niclosamide has been approved as anthelmintic against cestodes with well tolerated^9^ in humans for nearly 50 years.^10^ In recent years, niclosamide has been identified as a potential anti‐cancer agent in thyroid,^11^ renal,^12^ ovarian,^13^ lung,^14^ and prostate^15^ cancers. The downstream mechanism was also different in various cancers.^9^

We have synthesized and verified the compounds structures of niclosamide derivates with one substitution form A17 and ring fusion form B17. The cytotoxicity effect and mechanisms of these novel small molecular anti‐cancer drugs to tumor cells are clarified in this study.

## 2 MATERIAL AND METHODS

### 2.1 Chemical reagents and instruments in synthesis of niclosamide derivatives

All chemical reagents and solvents were purchased from Merck and Aldrich. The progress of the chemical reactions during niclosamide derivative synthesis was routinely checked by thin‐layer chromatography plates (Silica Gel F254 plates, Merck). ^1^H NMR and ^13^C NMR spectra of our synthetic compounds were determined with an Agilent 400 MR DD2 (400 MHz) apparatus. The melting points of all compounds were recorded with a Büchi 545 melting point apparatus. High‐resolution mass spectra of all compounds were obtained from Finnigan MAT 95S (high‐resolution electrospray ionization, HRESI) apparatus. The HPLC (model l‐2000, Hitachi) analysis was carried out using a C18 reverse‐phase column (XBridge BEH Shield RP18 Column, 130 Å, 5 mm, 4.6 mm × 250 mm, Waters) with UV detection (model l‐2400, Hitachi), and the mobile phase was methanol/water at a flow rate of 1.0 mL/min. A preliminary evaluation of the UV spectra of all compounds was recorded using methanol as a solvent, and the value of ƛmax for each compound was selected for the HPLC analysis.

### 2.2 Synthetic procedure: preparation of compound A17^16^

To solution of niclosamide (1.64 g, 5 mmol) and Zn dust (0.46 g, 7.08 mmol) in methanol (12.5 mL) were treated with HOAc (12.5 mL) slowly. A slight exotherm was noted during the early heating. Precipitate was observed in reaction mixture after 5 min and MeOH (5 mL) was added every 30 min for 1.5 hours to facilitate stirring. After 3 hours, the reaction mixture was filtered through a plug of Celite to remove the zinc and concentrated to afford an off‐white solid powder. The resulting solid was extracted with ethyl acetate and adjusted to PH = 8 with saturated NaHCO_3(aq)_. The organic layer was washed with brine, dried over MgSO_4_, and the solvent was concentrated in vacuo. The crude product was washed with CH_2_Cl_2_ and recrystallized from ethyl acetate/hexane to give the desired compound as a white powder.

### 2.3 Synthetic procedure: preparation of compound B17^17^

Methyl chloroformate (1 mL, 12 mmol) was added every 30 min within 3.5 hours dropwise with constant stirring to a solution of niclosamide (4 mmol) in anhydrous pyridine/THF (1:1, 40 mL) and the mixture was refluxed for 24 hours. After cooling to room temperature, the reaction mixture was poured into water (100 mL) and adjusted to pH = 6 with 1 N HCl_(aq)_, and the resulting solution was cooled in an ice bath. After 1 hour, the precipitate was collected and washed by CH_2_Cl_2_ to give the desired compound as a white powder.

### 2.4 Cell lines

Urothelial cell carcinoma (UCC) cell lines, J82, T24 (ATCC^®^ HTB‐1^™^ and ATCC^®^ HTB‐4^™^, Rockville, MD, USA) and TSGH2010, TSGH8301, and TSGH9202 (established in Uro‐Oncology laboratory, Tri‐Service General Hospital), renal cell carcinoma (RCC) cell lines, A498, ACHN, and Caki‐1 (ATCC^®^ HTB‐44^™^, CRL‐1611^™^, and HTB‐46^™^; Rockville), and prostate cancer (PCa) cell lines, DU145, LNCaP, and PC‐3 (ATCC^®^ HTB‐81^™^; ATCC^®^ CRL‐1740^™^, and CRL‐1435^™^, Rockville), hepatocellular carcinoma, Hep G2 (ATCC^®^ HB‐8065^™^, Rockville), mammary adenocarcinoma, MDA‐MB‐231 (ATCC^®^ HTB‐26^™^; Rockville), non‐small cell lung carcinoma, NCI‐H1299 (ATCC^®^ CRL‐5803^™^; Rockville), tongue squamous cell carcinoma, SCC‐25 (ATCC^®^ CRL‐1628^™^; Rockville), colorectal adenocarcinoma, HT‐29 (ATCC^®^ HTB‐38^™^; Rockville), and cervix squamous cell carcinoma, SiHa (ATCC^®^ HTB‐35^™^; Rockville) were cultured in Roswell Park Memorial Institute (RPMI) 1640 medium (Thermo Scientific, USA) supplemented with 10% fetal bovine serum (FBS) (Thermo Scientific), with 100 U/mL penicillin and 50 μg/mL streptomycin (Sigma, USA). The cells were grown at 37°C in a humidified 5% CO_2_ atmosphere. Confluent cells are detached with 0. 25% trypsin‐ethylenediaminetetraacetic acid (EDTA) (Sigma) before study or expansion.

### 2.5 MTT cytotoxic assay

Cellular chemosensitivity was assayed using a modified 3‐(4,5‐Dimethylthiazol‐2‐yl)‐2,5‐diphenyltetrazolium bromide (MTT) assay (Sigma) to determine cell viability in vitro. In brief, various cancer cell lines (2.5 × 10^3^ cells/well) in 100 μL culture medium were seeded into 96‐well microplates and incubated for 16 hours at 37°C before different agents exposure. The plated cell numbers were calculated to keep control cells growing in the exponential phase throughout the incubation period. Cells were treated with 100 μL of different agents and incubated for 72 hours. At that point, 100 μL of MTT (2 mg/mL in phosphate‐buffered saline (PBS) was added to each well and allowed to react for 3 hours. The blue formazan crystals formed were dissolved in 100 μL of dimethyl sulfoxide (DMSO) (Sigma). The optical density was determined by absorbance spectrometry at 560 nm using a microplate reader (Multiskan ex, Thermo Scientific). Three separate experiments with triplicate runs in each were performed to obtain mean cell viability. The drug concentrations inhibiting cell growth by 50% (IC_50_) was determined using the computer software Calcusyn 1.1 (Biosoft, Cambridge, UK).

### 2.6 Morphology monitoring

Cellular morphology was observed in culture microplate wells after different agent treatment under 100× power field light microscopy at 72 hours.

### 2.7 Scratch migration assay

Cell migratory ability was measured by scratch migration assay. Briefly representative cell lines with 2 × 10^5^ T24 and Caki‐1 cells, and 3 × 10^5^ DU145 cells were seeded on 24‐well microplates for 8 hours attachment, individually. The scratches were created by horizontal moved with 200 μL tips, washed with PBS twice, and refreshed the culture medium into different agent as the experiment start time point 0 hour. The diameter of scratch was recorded under light microscopy at 24 hours. The diameter was measured from each picture using SPOT software (USA). Each experiment was repeated at least three times independently.

### 2.8 Flow cytometric measurement of apoptosis and cell cycle changes

Annexin V and propidium iodide (PI, Sigma) stain was used for detection of early apoptosis changes. Briefly, 3 × 10^5^ T24 and Caki‐1 cells, and 5 × 10^5^ DU145 cells after different agent treatment for 72 hours were trypsinized and washed by cold PBS. Cells were stained by fluorescein‐conjugated annexin V **(**100 μg/mL**)** and PI (100 μg/mL) for 30 minutes at room temperature and then removed extract reagents. Cells were washed twice by cold PBS, suspended in 500 μL cold PBS, and analyzed by flow cytometer.

PI stain for cell cycle changes was performed as follows: 3 × 10^5^ T24, and Caki‐1 cells, and 5 × 10^5^ DU145 cells after different agent treatment for 72 hours were trypsinized and washed with PBS twice. Cells were harvested, washed twice by PBS, and fixed with 75% ethanol for 30 minutes. Then cells were washed twice by PBS and added 100 μg/mL RNase A (Sigma) for 15 minutes at room temperature. Cells were stained with 100 μg/mL of PI for 30 minutes at room temperature and then removed extract reagents. Cells were washed twice by PBS, suspended in 500 μL PBS, and analyzed by flow cytometer.

### 2.9 Western blotting detection of apoptosis changes

Western blotting was used to confirm the apoptosis‐related proteins changes with different antibodies against poly ADP‐ribose polymerase (PARP), caspase‐3, and bcl‐2, and alpha‐tubulins was used as loading control (Cell signaling, USA). Briefly, 3 × 10^5^ T24 and Caki‐1 cells, and 5 × 10^5^ DU145 cells after different agent treatment were trypsinized and washed with PBS twice. Cells were resuspended in 100 μL of RIPA buffer (Thermo Scientific) contained with cocktail protease inhibitor (Thermo Scientific). Twenty μg of extracted protein was electrophoresed for 2 hours in 12% SDS‐polyacrylamide gels and then transferred to polyvinylidene difluoride membranes (PVDF) (Millipore, USA) by electroblotter with 100 voltages for 1 hour at 4°C. Antibodies were diluted in Tris‐buffered saline with 0.1% Tween 20 (TBST) containing 5% non‐fat milk and membranes were incubated for 1 hour with gentle agitation. The blots were washed for three times with TBST and incubated with goat anti‐rabbit antibody conjugated to horseradish peroxidase for 1 hour. After three successive washes with TBST, Western blotting chemiluminescence reagent (Thermo Scientific) was used for protein detection. The membrane was pictured and analyzed by BioSpectrum 810.

### 2.10 Statistical analysis

Cytotoxicity, migratory ability, apoptotic changes, cell cycle, and proteins change were shown with mean ± standard deviation (SD). Student test was used for statistical comparison with differences considered significant at *P *<* *.05.

## 3 RESULTS

### 3.1 Basic characters of niclosamide derivatives, A17 and B17

**Figure 1:**
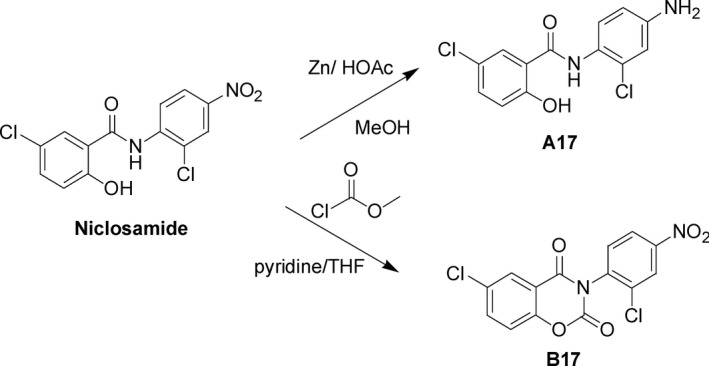
The scheme in synthesis of A17 and B17 derivatives from niclosamide

The chemical structure and molecular weight of niclosamide and its related derivatives A17 and B17 were as follow: niclosamide: 5‐Chloro‐N‐(2‐chloro‐4‐nitrophenyl)‐2‐hydroxybenzamide, molecular weight: 327 kDa; A17: N‐(4‐Amino‐2‐chlorophenyl)‐5‐chloro‐2‐hydroxybenzamide, molecular weight: 297 kDa; B17: 3‐(2‐Chloro‐4‐nitrolphenyl)‐6‐chloro‐2H‐benzo[e][1,3] oxazine‐2,4(3H)‐dione, molecular weight: 353 kDa (Figure 1). In preparation, B17 shows better solubility (10.6 mg/mL) than niclosamide (1.6 mg/mL) in DMSO.

### 3.2 Cytotoxicity of niclosamide, A17, and B17 on tested cancer cell lines

Various cancer cells in the exponential growth phases were treated with different doses of niclosamide, A17, and B17 for 72 hours, respectively (Table 1). The results showed that niclosamide and B17 inhibited cell growth in a dose‐dependent manner. A17 could not inhibit cell growth in lower concentration (<10 μmol/L). Among various cancer cell lines, the IC_50_ of niclosamide were 0.35 ± 0.09, 0.18 ± 0.05, 0.80 ± 0.37, 0.89 ± 0.33, 0.86 ± 0.30, 0.29 ± 0.10, 1.54 ± 0.17, 1.29 ± 0.11, 1.01 ± 0.09, 0.91 ± 0.28, 3.19 ± 0.37, 0.33 ± 0.07, 1.19 ± 0.20, 0.43 ± 0.09, 0.35 ±0.08, 1.08 ± 0.11, and 0.76 ± 0.22 μmol/L on J82, T24, TSGH2010, TSGH8301, TSGH9202, A498, ACHN, Caki‐1, DU145, LNCaP, PC‐3, Hep G2, HT‐29, MDA‐MB‐231, NCI‐H1299, SCC‐25, and SiHa cells individually. The IC_50_ of B17 were 0.41 ± 0.10, 0.10 ± 0.05, 0.86 ± 0.38, 0.97 ± 0.35, 0.86 ± 0.29, 0.47 ± 0.21, 3.36 ± 0.21, 1.76 ± 0.12, 1.15 ± 0.10, 0.90 ± 0.26, 3.77 ± 0.37, 0.28 ±0.08, 1.04 ± 0.12, 0.28 ± 0.08, 0.25 ± 0.08, 0.85 ± 0.27, and 0.59 ± 0.18 μmol/L of J82, T24, TSGH2010, TSGH8301, TSGH9202, A498, ACHN, Caki‐1, DU145, LNCaP, PC‐3, Hep G2, HT‐29, MDA‐MB‐231, NCI‐H1299, SCC‐25, and SiHa cells individually.

**Table 1:** ICs of niclosamide and it derivatives A17 and B17 on various cancer cell lines

Cell line	Niclosamide	A17	B17
(μmol/L)	IC_50_	IC_50_	IC_50_
J82	0.35 ± 0.09	>10	0.41 ± 0.10
T24	0.18 ± 0.05	>10	0.10 ± 0.05
TSGH2010	0.80 ± 0.37	>10	0.86 ± 0.38
TSGH8301	0.89 ± 0.33	>10	0.97 ± 0.35
TSGH9202	0.86 ± 0.30	>10	0.86 ± 0.29
A498	0.29 ± 0.10	>10	0.47 ± 0.21
ACHN	1.54 ± 0.17	>10	3.36 ± 0.21
Caki‐1	1.29 ± 0.11	>10	1.76 ± 0.12
DU145	1.01 ± 0.09	>10	1.15 ± 0.10
LNCaP	0.91 ± 0.28	>10	0.90 ± 0.26
PC‐3	3.19 ± 0.37	>10	3.77 ± 0.37
Hep G2	0.33 ± 0.07	ND	0.28 ± 0.08
HT‐29	1.19 ± 0.20	ND	1.04 ± 0.12
MDA‐MB‐231	0.43 ± 0.09	ND	0.28 ± 0.08
NCI‐H1299	0.35 ± 0.08	ND	0.25 ± 0.08
SCC‐25	1.08 ± 0.11	ND	0.85 ± 0.27
SiHa	0.76 ± 0.22	ND	0.59 ± 0.18

Among these urological cancers, T24 is a metastatic UCC cell line and has better drug sensitivity to niclosamide and B17. Caki‐1 and ACHN are metastatic RCC and Caki‐1 has better drug sensitivity to niclosamide and B17. DU145 and PC‐3 are androgen resistant PCa^18^ and DU145 has better drug sensitivity to niclosamide and B17. Hence, we choose T24, Caki‐1, and DU145 as the representative cell lines for further study.

### 3.3 Morphological changes of tumor cells after niclosamide and B17 treatment

**Figure 2:**
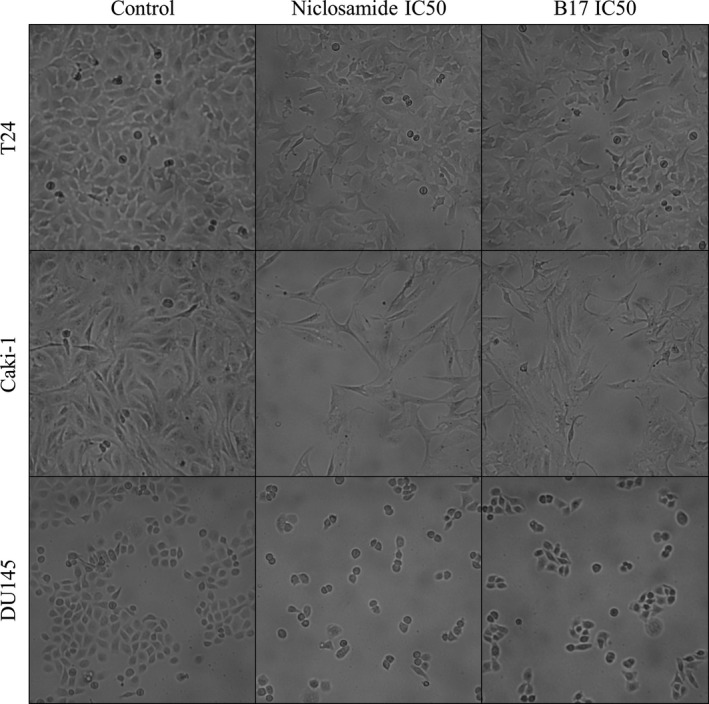
Morphology changes after niclosamide and B17 treatment on T24, Caki‐1, and DU145 cells at 72 hours under 100× power field light microscope

T24, Caki‐1, and DU145 cells were treated byIC_50_ of niclosamide and B17 individually for 72 hours (Figure 2). In T24 UCC cells, both niclosamide and B17 treatment showed cells transformed into polygonal shape with less mitotic activity when compared to control cells but no prominent apoptotic body was seen. In Caki‐1 RCC cells, both niclosamide and B17 treatment showed cells transformed spindle shape with less mitotic cells as compared to control cells but no prominent apoptotic body was seen. In DU145 PCa cells, both niclosamide and B17 treatment showed cells transformed into round shape with less mitotic cells as compared with control cells but no prominent apoptotic body was seen too. These results indicate that in general cancer cells growth were inhibited without prominent apoptotic body formation in early phase after niclosamide and B17 treatment in T24, Caki‐1, and DU145 cells.

### 3.4 Migratory inhibition of tumor cells after niclosamide and B17 treatment

**Figure 3:**
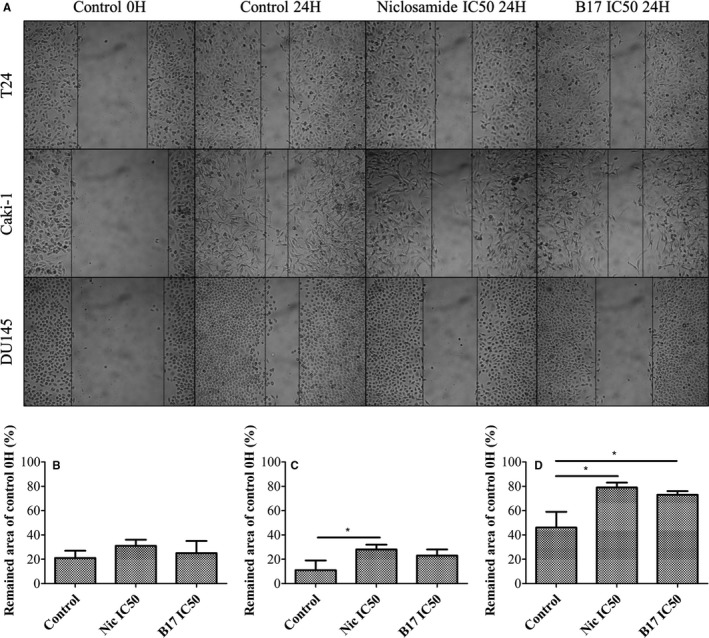
Scratch migration assay of (A) migratory abilities changes after niclosamide and B17 treatment on T24, Caki‐1, and DU145 cells at 24 hours under 100× power field light microscopy. Statistical analysis was showed as histogram graph of (B) T24, (C) Caki‐1, and (D) DU145 cells, respectively (Student *t*‐test, *P <* *.05, error bars = SD)

T24, Caki‐1, and DU145 cells were treated by IC_50_ of niclosamide and B17 for 24 hours on culture dishes. The observed remained area was slightly increased after niclosamide (30.8 ± 5.4%, *P *=* *.100) and B17 (24.5 ± 9.8%, *P *=* *.650) treatment when compared to control group (21.3 ± 5.5%) in T24 UCC cells (Figure 3). The observed remained area dramatically increased after niclosamide (28.5 ± 3.5%, *P *=* *.032) treatment but only moderately increased in B17 treatment (22.7 ± 5.3%, *P *=* *.123) when compared to control group (11.5 ± 8.4%) in Caki‐1 RCC cells. Interestingly, the observed remained area dramatically increased after niclosamide (78.7 ± 3.7%, *P *=* *.013) and B17 (72.8 ± 2.7%, *P *=* *.024) treatment when compared to control group (45.9 ± 12.8%) of DU145 PCa cells. These results indicate that niclosamide has significant inhibitory effect on migratory activity of tumor cells while B17 has moderate inhibitory activity only. Among them, DU145 tumor cells are most sensitive to niclosamide and B17 treatment in migratory inhibition.

### 3.5 Apoptosis changes of tumor cells after niclosamide and B17 treatment

**Figure 4:**
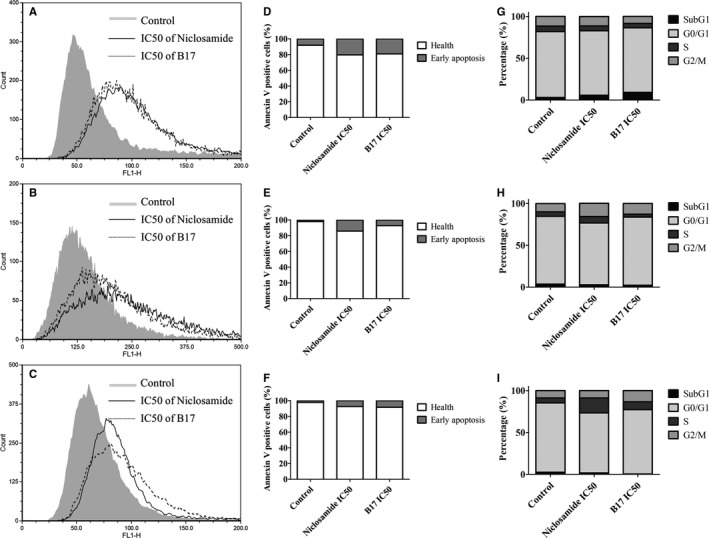
Flow cytometry analyses of (A‐C) annexin V stain, (D‐F) histogram graph and (G‐I) cell cycle changes after niclosamide IC50 and B17 IC50 treatment on T24, Caki‐1, and DU145 cells at 72 hours, respectively

The annexin V staining has been used to detect the apoptotic cells after individually IC_50_ of niclosamide and B17 treatment for 72 hours in T24, Caki‐1, and DU145 cells. The apoptotic cells were slightly increased after niclosamide (20.4%) and B17 (19.2%) treatment than control group (8.1%) in T24 UCC cells (Figure 4). Meanwhile, apoptotic cells were slightly increased after niclosamide (14.1%) and B17 (7.2%) treatment than control group (2.0%) in Caki‐1 RCC cells, and they were slightly increased after niclosamide (7.4%) and B17 (8.4%) treatment than control group (2.2%) in DU145 PCa cells. These results indicate that both niclosamide and B17 can induced apoptotic cell occurrence in UCC, RCC, and PCa cells while UCC cells had the highest changes than RCC and PCa cells.

### 3.6 Cell cycle changes of tumor cells after niclosamide and B17 treatment

The PI stain was used to measure the cell cycle changes after individually IC_50_ of niclosamide and B17 treatment for 72 hours in T24, Caki‐1, and DU145 cells. The percentage of apoptotic body (SubG1 phase) was slightly increased after niclosamide (5.7%) and B17 (9.1%) treatment when compared to control (3.1%) in T24 cells (Figure 4G‐I). The SubG1 phase was not significantly changed after niclosamide (2.8%) and B17 (2.2%) treatment when compared to control (3.6%) in Caki‐1 RCC cells, and it was not significantly changed after niclosamide (1.7%) and B17 (0.5%) treatment when compared to control (2.6%) in DU145 PCa cells too. Cell cycle was not significantly changed at G0/G1, S, or G2/M phases after niclosamide and B17 treatment in T24, Caki‐1, and DU145 cells. These results indicate that only T24 UCC cells been activated the programmed cell death process after niclosamide and B17 treatment.

### 3.7 Apoptosis‐ and survival‐related proteins changes in tumor cells after niclosamide and B17 treatment

**Figure 5:**
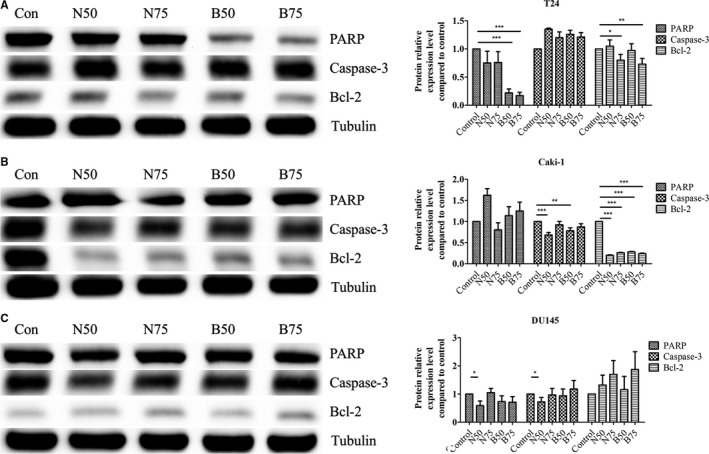
Western blotting analysis of apoptosis‐related proteins change of PARP, caspase‐3, and bcl‐2 in (A) T24, (B) Caki‐1, and (C) DU145 cells. Exponentially growing cells were treated by IC_50_ and IC_75_ of niclosamide and B17 for 72 hours. Alpha‐tubulin was used for normalization and verification of protein loading. Statistical analysis was showed as histogram graphs of (D) T24, (E) Caki‐1, and (F) DU145 cells, respectively (Student *t*‐test, *P <* *.05, **P <* *.01, and ***P <* *.001, error bars = SD)

The anti‐apoptosis protein bcl‐2 and pro‐apoptosis proteins PARP and caspase‐3 were used to confirm the mechanism of cell death after individually niclosamide and B17 treatment in T24, Caki‐1, and DU145 cells. PARP expression was dramatically decreased in both of IC_50_ and IC_75_ of B17 treatment (0.22 ± 0.07, *P *<* *.001 and 0.17 ± 0.06, *P *<* *.001) than IC_50_ and IC_75_ of niclosamide (0.75 ± 0.21, *P *=* *.108 and 0.76 ± 0.19, *P *=* *.100) treatment and control cells in T24 UCC cells (Figure 5A‐F). Bcl‐2 was only decreased in IC_75_ (0.80 ± 0.10, *P *=* *.024 and 0.73 ± 0.10, *P *=* *.009) but not IC_50_ (1.05 ± 0.11, *P *=* *.444 and 0.97 ± 0.12, *P *=* *.702) of niclosamide and B17 treatment in T24 cells. These results indicate that the program cell death is activated through PAPR protein after higher dose of B17 treatment in T24 cells.

Caspase‐3 expression was dramatically decreased in IC_50_ of niclosamide (0.68 ± 0.06, *P *<* *.001) and IC_50_ and IC_75_ of B17 (0.78 ± 0.07, *P *=* *.006 and 0.87 ± 0.08, *P *=* *.044) than IC_75_ of niclosamide (0.92 ± 0.08, *P *=* *.131) treatment and control for 72 hours in Caki‐1 RCC cells. Bcl‐2 was dramatic decreased in IC_50_ and IC_75_ of niclosamide (0.20 ± 0.01, *P *<* *.001 and 0.26 ± 0.01, *P *<* *.001) and IC_50_ and IC_75_ of B17 (0.28 ± 0.01, *P *<* *.001 and 0.24 ± 0.01, *P *<* *.001) than control cells in Caki‐1 cells. These results indicate that the program cell death was majorly activated through bcl‐2 protein after niclosamide and B17 treatment in Caki‐1 RCC cells.

PARP and caspase‐3 expression were only decreased in IC_50_ of niclosamide (0.59 ± 0.16, *P *=* *.011 and 0.72 ± 0.08, *P *=* *.003) treatment when compared to control group in DU145 PCa cells. These results suggest that the program cell death was activated through PAPR and caspase‐3 protein simultaneously after niclosamide treatment in DU145 PCa cells.

## 4 DISCUSSION

Among urological cancers, urothelial carcinoma, renal cell carcinoma, and prostatic carcinoma are three prevalent tumors worldwide. Despite conventional therapies such as surgery, radiation, and chemotherapy, the overall survival rate for these cancers still need improving although target therapy and immunotherapy are emerging as new therapies with increasing survival in urothelial carcinoma and renal cell carcinoma.^19^, ^20^, ^21^ Multidrug resistance after chemotherapy in advanced urothelial carcinoma and prostate carcinoma and hormonal resistance in metastatic prostate cancer usually are main obstacles for sustained treatment.^22^, ^23^, ^24^, ^25^ Therefore, more effective approaches need to be developed to improve the treatment and prognosis of these cancers.

Many studies have reported on the niclosamide enhances cytotoxicity on various cancers, including oral cancer, lung cancer, colon, thyroid, cervix, ovarian, kidney, and prostate cancers, via STAT3, EGFR/PI3K/Akt, RANKL Wnt, mTOR, HIF‐1α/VEGF, Ras, c‐myc, and Notch signal pathways and mitochondria dysfunction.^11^, ^12^, ^13^, ^14^, ^26^, ^27^, ^28^, ^29^, ^30^, ^31^, ^32^, ^33^ Cancer cell upregulates other important downstream genes such as STAT3, which contributes to cell proliferation, cell survival, and angiogenesis in many cancers. It has been reported that multiple signal transduction pathway inhibitors, including the mitogen‐activated protein kinase, fibroblast growth factor receptor inhibitors, and chemotherapy drugs, can induce activation of STAT3 survival signaling pathway, leading to drug resistance.^34^, ^35^ Our study revealed that PARP and bcl‐2 apoptotic signaling pathway may be involved in the cytotoxicity of B17 niclosamide derivative for urological cancer, especially the urothelial carcinoma cells.

Here, our results in this study showed that niclosamide and synthesized derivative, B17, reduce cell viability of urological cancer cell lines in a dose and time‐dependent manner. This finding suggests that niclosamide and B17 could be effective against urological cancers. The cytotoxic ability of niclosamide and its derivative B17 presented acceptable results to these tested urological cell lines, including urothelial carcinoma, renal cancer, and prostate cancer. Among them, urothelial carcinoma cells have highest sensitivity to niclosamide and B17 treatment when compared to renal cancer and prostate cancer. These tumor cells had decreased mitotic activity without morphological changes within 72 hours under niclosamide and B17 treatment.

Regarding influence on migratory activity of tumor cells, prostate cancer cells had most prominent inhibition by niclosamide and B17 treatment on cellular migration than renal cancer and urothelial cancer. Interestingly, it indicates that niclosamide and B17 conducted their cytotoxic effect on urological cancers through different mechanisms and migratory activity inhibition may not one major part of cellular cytotoxicity conducted by niclosamide and B17.

Further evaluation on cell cycle effect by niclosamide and B17, we found that both of them have significant increasing in early apoptosis and sub‐G1 cells of urothelial carcinoma than renal and prostate cancer cells. As mediated pathway of apoptosis, marked increased PARP and bcl‐2 proteins were seen in urothelial cancer cells while increased caspase‐3 and bcl‐2 were seen in renal cancer cells. These results may indicate that niclosamide and B17 conduct the immediate cytotoxic effect on urothelial cancer cells through PARP and bcl‐2 activation pathway with occurrence of early apoptosis and sub‐G1 cell arrest. On the contrary, they conduct the delayed suppression effect on renal cancer and prostate cancer growth via other pathways, including caspase‐3 activation, with migration inhibition. Apoptosis of tumor cells can be induced by the activation of the PARP‐bcl‐2‐caspase 3 pathway, which also is related to mitochondria activity.^36^, ^37^, ^38^ Different urological cancers present different sensitivity and related cytotoxicity by niclosamide and B17 derivative via PARP‐bcl‐2 signal pathway in this study. Nevertheless, niclosamide and B17 may conduct their cytotoxicity through other signal pathways and mitochondria alternation.

Drug development process from initial lead discovery to final medication is costly, lengthy, and incremental. Finding new uses for old or natural products is much easier and more economical than inventing a new drug from scratch. Niclosamide is practically insoluble in water (230 ng/mL), which may severely limit its efficacy in other field application, such as intravascular or intracavitary routes.^39^ Previous study showed that niclosamide derivatives increased both plasma concentration and the duration.^17^ Further, niclosamide containing salicylanilide core structure showed various biological activities, and ring fusion form of salicylanilide had higher biological activity in inhibition of osteoclastogenesis.^40^

In summary, the B17, one newly synthetic derivative of niclosamide in our laboratory showed increased solubility than native niclosamide and stable pharmacodynamics in rat (data not shown). The present study demonstrated that B17 can inhibit the cell growth of urological cancers by inhibition of PARP‐bcl‐2 pathway with induction of apoptosis and it may have the potential been used as a new alternative intravesical treatment agent for superficial bladder cancer or adjuvant reagent during chemotherapy for overcoming the drug resistance generation.

## DISCLOSURE STATEMENT

The authors do not have any conflict of interests.
